# The global flexible and navigable suction ureteral access sheaths (FANS) survey on utility, practices and future needs in flexible ureteroscopy: a EAU Endourology, PEARLS and IAU collaboration

**DOI:** 10.1007/s00345-026-06321-4

**Published:** 2026-03-10

**Authors:** Steffi Kar Kei Yuen, Bhaskar Somani, Amelia Pietropaolo, Daniele Castellani, Wen Zhong, Wei Zhu, Arman Tsaturyan, Jia-Lun Kwok, Michael Wong, Tzevat Tefik, Yiloren Tanidir, Satyendra Persaud, Wissam Kamal, Boyke Soebhali, Deepak Ragoori, Mohamed Elshazly, Anil Shrestha, Ben Hall Chew, Chi-Fai Ng, Guohua Zeng, Vineet Gauhar

**Affiliations:** 1 S.H. Ho Urology Centre, Department of Surgery, The Chinese University of Hong Kong, Hong Kong, China; 2 European Association of Urology Section of Endourology (ESEUT), Arnhem, The Netherlands; 3 Department of Urology, University Hospital Southampton, NHS Trust, Southampton, UK; 4 Urology Unit, Azienda Ospedaliero-Universitaria Delle Marche, Via Conca 71, Ancona, 60126 Italy; 5 Department of Urology, The First Affiliated Hospital of Guangzhou Medical University, Guangzhou, China; 6 Department of Urology, Shufu People’s Hospital, Kashgar Prefecture, Xinjiang Uygur Autonomous Region China; 7 Department of Urology, Erebouni Medical Center, Yerevan, Armenia; 8 Department of Urology, Yerevan State Medical University, Yerevan, Armenia; 9 Department of Urology, Tan Tock Seng Hospital, Singapore, Singapore; 10 Department of Urology, Singapore General Hospital, Singapore, Singapore; 11 Department of Urology, Istanbul University Istanbul Faculty of Medicine, Istanbul, Turkey; 12 Department of Urology, Medicana Atasehir Hospital, Istanbul, Türkiye Turkey; 13 Division of Clinical Surgical Sciences, University of the West Indies, St. Augustine, Trinidad and Tobago; 14 Urology Unit, King Fahd General Hospital, Jeddah, Saudi Arabia; 15 Department of Urology, Abdul Wahab Sjahranie Hospital Medical Faculty, Muliawarman University, Samarinda, Indonesia; 16 Department of Urology, Asian Institute of Nephrology & Urology, Irram Manzil Colony, Hyderabad, Telangana India; 17 Urology Unit, Menoufia University, Shibin el Kom, Egypt; 18 Department of Urology, National Academy of Medical Sciences, Bir Hospital, Kathmandu, Nepal; 19 Department of Urology, University of British Columbia, Vancouver, Canada; 20 Department of Urology, Ng Teng Fong Hospital, Singapore, Singapore

**Keywords:** Survey, Suction, Flexible and navigable suction ureteral access sheath, Flexible ureteroscopy, Urolithiasis

## Abstract

**Introduction:**

The landscape of flexible ureteroscopy (fURS) is evolving with the use of flexible and navigable suction ureteral access sheaths (FANS) as surgeons gain experience utilizing suction. Surveys offer a unique lens through which to evaluate the adoption, efficacy, and challenges associated with new endourology technology. As FANS become widespread, we aimed to capture real-world practice patterns of FANS utilization in fURS.

**Methods:**

A collaborative effort by the European Association of Urology (EAU), Progressive Endourological Association for Research and Leading Solutions (PEARLS) and International Alliance of Urolithiasis (IAU) lead to the design and dissemination of a 41-question survey via SurveyMonkey and social media from 19 April to 29 May 2025. Participation was voluntary. Descriptive statistics were applied to demographic details and categorical responses.

**Results:**

A total of 680 participants had completed the questionnaire. The majority of respondents were endourology fellowship-trained consultants (71.8%) practicing in teaching hospitals. Overall, 91% of urologists used suction in fURS, with 28.7% having replaced all conventional sheaths with FANS. The most common indications included multiple renal stones, stones 1–2 cm in diameter, and bilateral stones. Key technical challenges included suction-induced pelvicalyceal system collapse (51.9%) and lower pole access (37.6%). Notably, 39.9% were unaware of their irrigation flow rates, and 28.1% did not know suction pressure settings. Low-power Holmium laser with a dusting-fragmentation-aspiration strategy was preferred. Future development priorities included defining irrigation/suction parameters (29.6%) and integrating pressure control mechanisms (25.9%).

**Conclusion:**

This survey highlights the rapid global adoption of FANS and its expanding role in complex stone management. Despite its perceived benefits in stone clearance and infection reduction, significant technical variability and knowledge gaps persist, underscoring the need for standardized protocols, structured training, and further technological innovation to optimize clinical implementation.

**Supplementary Information:**

The online version contains supplementary material available at 10.1007/s00345-026-06321-4.

## Introduction

Surveys play an increasingly vital role in understanding current surgical practices by identifying trends in new technical innovations and assessing outcomes within specialized medical fields, urologists seek to use these as information gathering tools to improve practice [1]. In endourology subspecialty focused surveys on minimally invasive surgical procedures like percutaneous nephrolithotomy, webinar based learning and their utility is time tested [2].

The landscape of flexible ureteroscopy (fURS) evolves with the use of flexible and navigable suction ureteral access sheaths (FANS) as surgeons gain experience utilizing suction. The clinical advantages of suction in fURS and laser lithotripsy has been well established [3] and as these advanced techniques become widespread for managing urolithiasis, surveys provide invaluable insights into surgeon preferences, equipment utilization and the effectiveness of different treatment algorithms [4, 5].

Surveys of diverse practitioners provide valuable real-world insights into the adoption, efficacy, and challenges associated with fURS and laser lithotripsy, as well as their role in optimizing patient care and advancing endourological practice [6]. Amongst suction technologies in fURS, FANS is now an established game changer entity in normal renal anatomy when used by expert surgeons with proven higher stone-free rates, reduced post-operative infection rates and stone basket usage and better quality of life [7, 8], leading some surgeons to adopt these suction access sheaths exclusively [9].

The present study was conducted as a collaborative effort by European Association of Urology (EAU), Progressive Endourological Association for Research and Leading Solutions (PEARLS) and International Alliance of Urolithiasis (IAU). Through conducting this global survey, we invited surgeons using FANS in their real world practice to opine on aspects pertaining to intraoperative and perioperative steps, pros and cons and future needs, which could help guide the development of best practice policies.

## Material and methods

### Aim

The primary outcome of the survey was to assess current practices with FANS in flexible ureteroscopy for stones.

### Overview

A customized online questionnaire was developed to assess urologists’ practices on utilization of FANS, operative and technical considerations, surgical strategies and preferences, challenges encountered and vision for future development of FANS in flexible ureteroscopy. The survey was opened to all with an intention of conducting a cross-sectional analysis of consultants, attending and urologists in training. All participation was voluntary.

### Questionnaire design

A systematic review of the existing literature was first conducted. The first draft of the survey questionnaire was formulated after extracting insights from the literature review by two authors (SY and VG). Structured interviews were conducted with five endourologists who perform more than 200 flexible ureteroscopy with FANS annually for critical analysis of the draft. The refined questionnaire was further modified after circulation with final approval seek from the EAU Endourology and IAU committee board (BS, AP, ZG) prior to public circulation on social media domains such as #UroSoMe X platform. Prior to public circulation of the survey, the finalized survey was circulated electronically and tested by ten urologists not involved with the survey design to ensure questionnaire clarity and feasibility of survey completion.

The finalized questionnaire consisted of 41 questions encompassing the following 5 domains: (Supplementary Appendix)

1. Demographic information, clinical practice and experience with FANS flexible ureteroscopy (questions 1–17).
2. Indication of FANS in one’s own practice (questions 18–21).
3. Perioperative and operative strategies with FANS flexible ureteroscopy (questions 22–34).
4. Exit strategy and protocol for follow-up imaging (questions 35–38).
5. Challenges encountered in the utility of FANS and vision for future development (questions 39–41).

The questionnaire, hosted on SurveyMonkey, was restricted to one response per IP address and disseminated globally via social media platforms like X from 19 April to 29 May 2025. All participations were voluntary. The study was conducted in compliance with the Declaration of Helsinki. Ethical review was waived for this anonymous survey, and informed consent was implied by the participants’ voluntary completion of the questionnaire.

### Data synthesis and analysis

A total of 801 responses were received, of which 680 participants had completed the questionnaire. Responses with missing information were excluded for analysis. Descriptive statistics were applied to demographic details and categorical responses using SPSS software v20.0.

## Results

### Background demographics

**Fig. 1:**
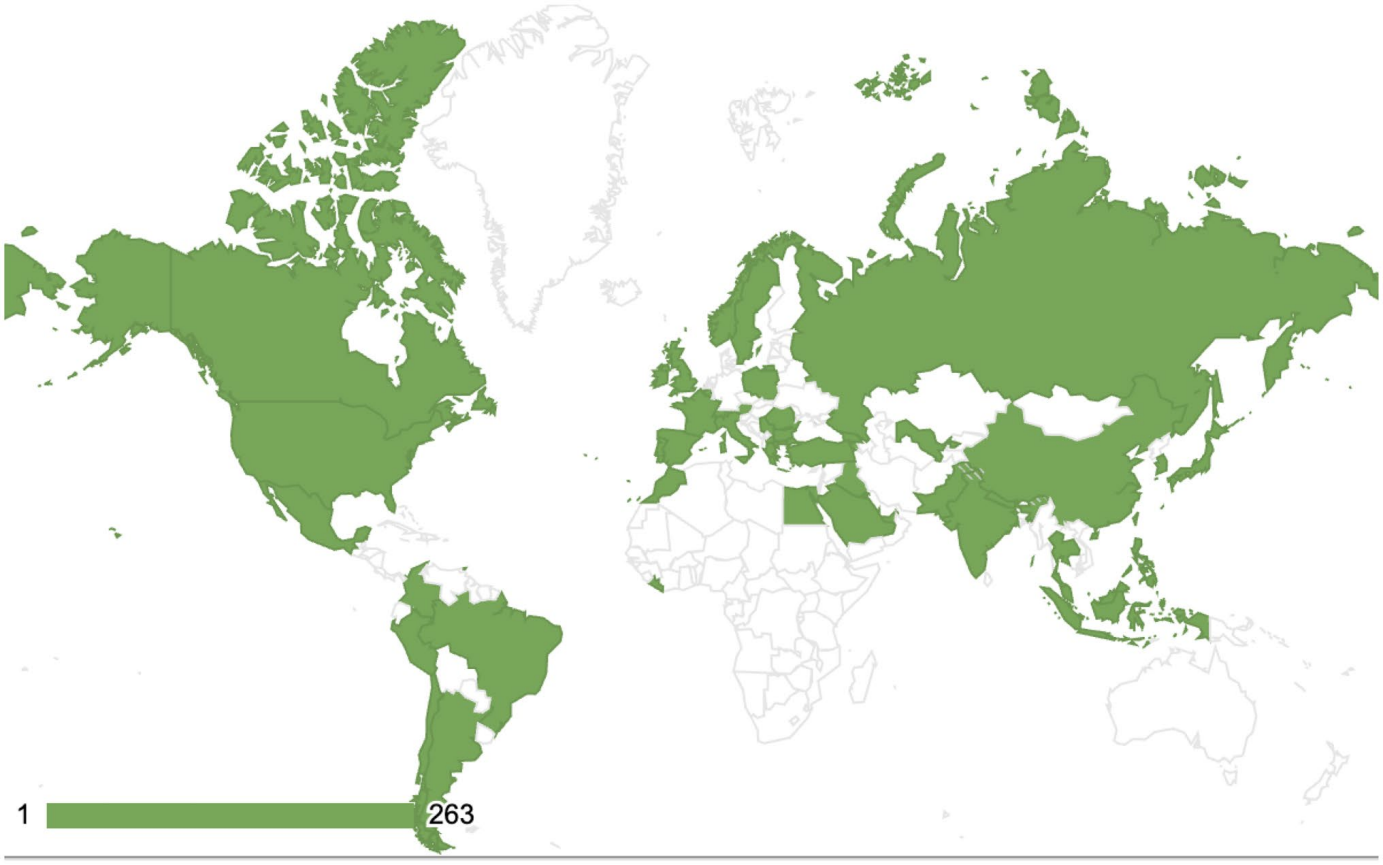
Heatmap distribution of global survey participants (green – indicate participants; white – no participants)

Respondent demographics are summarized in Table 1. Majority of the respondents are consultants (82.4%), endourology fellowship trained (71.8%), practising in teaching hospitals with residents (56.2%). The highest five countries of survey participants are from China, followed by Turkey, India, Egypt and the Philippines (Supplementary Table). Figure 1 shows the heatmap distribution of global survey participants.

### Clinical practice and experience with FANS flexible ureteroscopy

Overall, 91% reported using suction in flexible ureteroscopy with 29.1% choosing FANS in at least a quarter of their cases. A total of 195 (28.7%) have reported to replace all conventional ureteral access sheaths with FANS in their routine practice. While using the FANS sheath, 490 (72.1%) required an assistant. Most common reason was to help in holding the sheath (41.4%); followed by helping with vent regulation to control suction (38.2%).

**Table 1:** Survey respondents’ demographics

	*N* = 680	Percentage
Gender		
- Male	644	92.8%
- Female	36	7.2%
Age group		
- < 30 years - 31–40 years - 41–50 years - > 50 years	56 202 250 172	8.2% 29.7% 36.8% 25.3%
Healthcare system		
- University teaching hospital with residents - Private/corporate hospital - Public hospital/ No residents - Both public and private	382 78 176 44	56.2% 11.5% 25.9% 6.5%
Position held		
- Consultant - Fellow - Resident	560 67 53	82.4% 9.9% 7.8%
Endourology fellowship trained		
- Yes - No	488 192	71.8% 28.2%
Years of experience as a urologist		
- < 10 years - 10–20 years - > 20 years - In training	152 224 278 26	22.4% 32.9% 40.9% 3.8%
Case volume per annum of fURS in department		
- < 10 - 11–50 - 51–100 - 101–200 - > 200	36 84 99 146 315	5.3% 12.4% 14.6% 21.5% 46.3%
Proportion of fURS utilizing FANS		
- < 25% - 25–50% - 50–75% - Almost all - None	198 103 134 195 50	29.1% 15.1% 19.7% 28.7% 7.4%
Proportion of fURS utilizing conventional ureteral access sheath		
- < 25% - 25–50% - 50–75% - Almost all - None	187 119 119 136 119	27.5% 17.5% 17.5% 20.0% 17.5%
Proportion of fURS utilizing suction		
- < 25% - 25–50% - 50–75% - Almost all - None	170 105 133 211 61	25.0% 15.4% 19.6% 31.0% 9.0%
Proportion of fURS that utilized conventional ureteral access sheath converted to FANS		
- < 25% - 25–50% - 50–75% - Almost all - None	213 66 49 107 245	31.3% 9.7% 7.2% 15.7% 36.0%
Monthly case load of FANS flexible ureteroscopy in the department		
- < 10 - 11–15 - 16–20 - > 20 - > 50	259 100 77 105 139	38.1% 14.7% 11.3% 15.4% 20.4%
Personal monthly case load of FANS flexible ureteroscopy with role as chief surgeon or first assistant		
- < 10 - 11–15 - 16–20 - > 20 - > 50	231 149 120 109 71	34.0% 21.9% 17.6% 16.0% 10.4%
Assistant required routinely during FANS flexible ureteroscopy		
- None - 1 assistant - More than 1 assistant	169 490 21	24.9% 72.1% 3.1%
Role of assistant (if required)		
- Help stabilize FANS - Help suction by regulating the suction vent - Other reasons	206 190 102	41.4% 38.2% 20.5%
Antibiotic regime*		
- Intraoperative single dose antibiotics in all and treat any positive culture - Treat only if culture positive - Postoperative antibiotics only if required - Mandatory routine postoperative antibiotics	476 70 101 249	70.0% 10.3% 14.9% 36.6%

*question allowed multiple answers

### Indication of FANS in one’s own practice

**Fig. 2:**
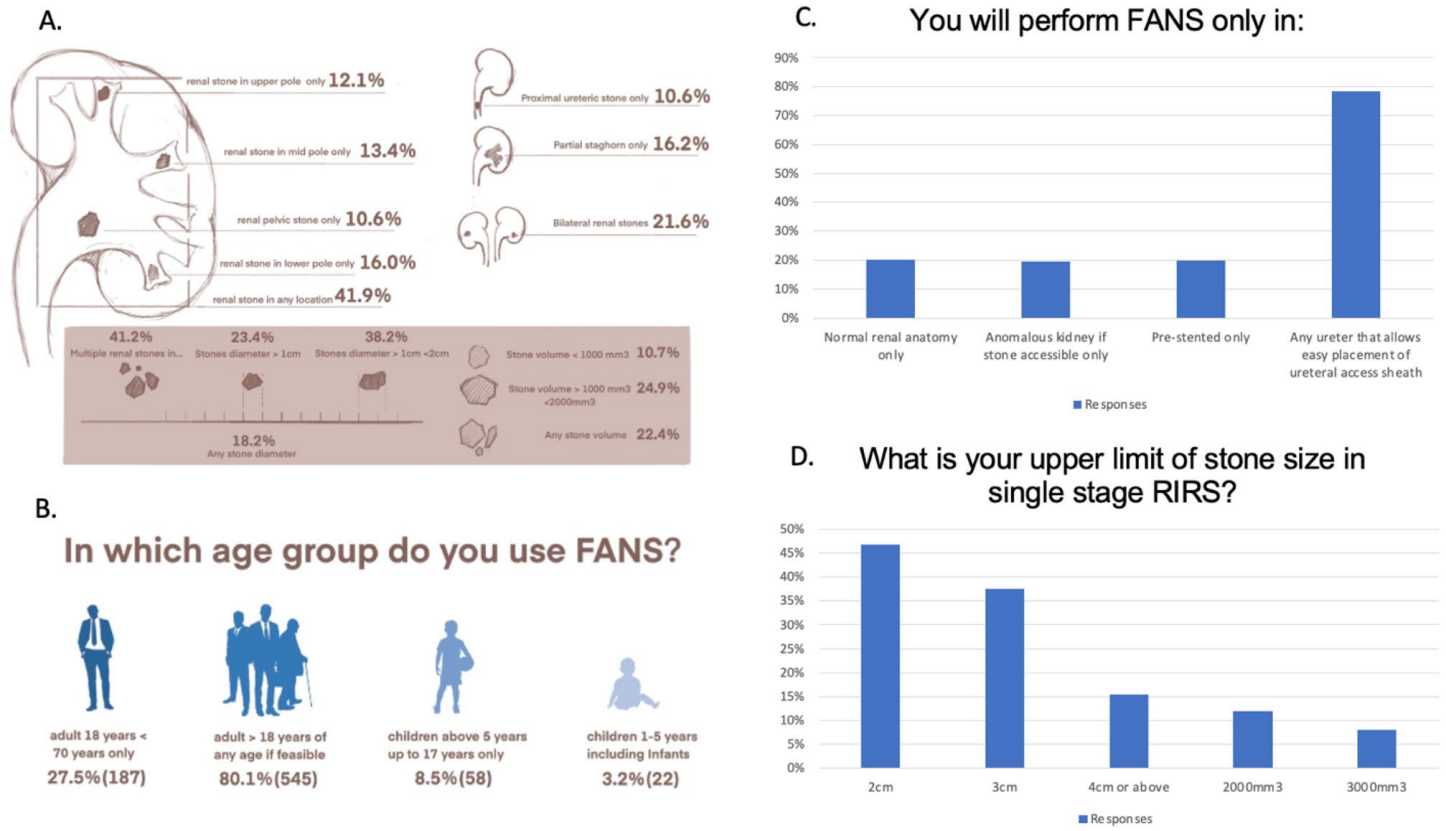
**A** Clinical indications for FANS;** B** Age considerations for FANS;** C** Preference in FANS utilization;** D** Upper limit of stone size and volume for FANS. FANS – flexible and navigable suction ureteral access sheath; RIRS – retrograde intrarenal surgery

The most common indications for FANS include multiple renal stones in multiple or any locations, stone diameter of 1–2 cm, stone volume of 1000-2000mm^3^, and bilateral renal stones (Fig. 2A). Only a minority had used FANS in children aged 5–17 years and even fewer for 1–5 years. Most preferred to use in adults aged 18–70 years(Fig. 2B).

A total of 134 respondents (19.7%) chose prestenting (Fig. 2C) and 318(46.8%) used 2 cm stone diameter as the cutoff for in single stage FANS fURS. Furthermore, 256 (37.6%) were confident of using FANS even with a 3 cm stone (Fig. 2D).

### Perioperative and operative strategies with FANS flexible ureteroscopy

#### Procedural steps and mode of anaesthesia

**Fig. 3:**
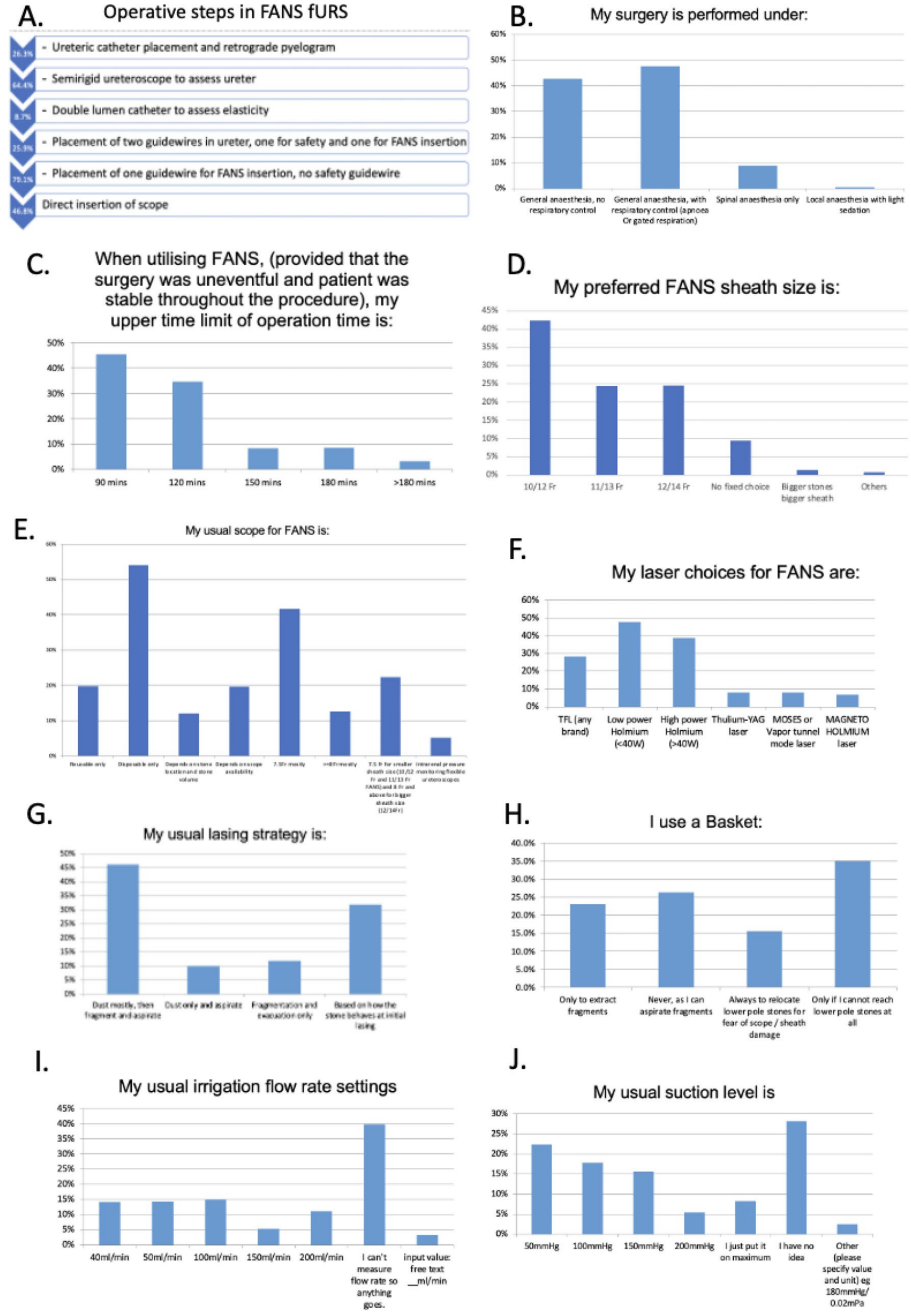
**A** Preference in procedural steps for FANS fURS; **B** Usual mode of anesthesia in FANS fURS; **C** Upper time limit of operation time when utilizing FANS; **D** Preferred FANS sheath size; **E** Preferred flexible ureteroscope choice; **F** Preferred laser choices for FANS flexible ureteroscopy; **G** Preferred lasing strategy in FANS fURS; **H** Basket utilization; **I** Preferred irrigation flow rate settings; **J** Preferred suction settings

A total of 438 respondents (64.4%) reported the routine use of semirigid ureteroscopy to assess the ureter status prior to FANS insertion; whilst 318 (46.8%) proceed directly insertion of FANS and flexible ureteroscope. Only 176 (25.9%) utilize a safety guidewire routinely during FANS flexible ureteroscopy (Fig. 3A). Most (90.4%) performed FANS flexible ureteroscopy under general anaesthesia with or without respiratory control (Fig. 3B). Overall, 45.4% followed the 90 min upper time limit for flexible ureteroscopy when utilizing FANS. However, 34.6% pushed the upper limit to 120 min (Fig. 3C).

#### Sheath, scope and laser preferences

10/12Fr is the most common choice of FANS sheath size (Fig. 3D). Disposable flexible ureteroscopy are more commonly used with FANS (51.4%). The most common scope size is 7.5 Fr (41.7%) (Fig. 3E). Usual laser choices for FANS are low power Holmium (47.6%), followed by high power Holmium (38.8%), and thulium fiber laser (28.2%) (Fig. 3F). The most common lasing strategy is dusting mostly, followed by fragmentation and aspiration (46.2%) (Fig. 3G). 26.3% do not routinely use stone baskets, except when unable reach lower pole stones (35.0%); relocate lower pole stones for fear of scope/ sheath damage (15.6%). (Fig. 3H)

#### Irrigation and suction devices and settings preferences

Most common irrigation flow rate and suction settings were 100 ml/min (15.0%) and 50mmHg (22.4%) respectively. However, 39.9% were not aware of the actual irrigation flow rate (Fig. 3I); 28.1% do not know the actual suction pressure applied on FANS (Fig. 3J). Common irrigation techniques include irrigation pumps (41.1%); gravity with pressurized bag (24.3%); gravity alone (18.8%) and automated irrigation suction systems with intrarenal pressure monitoring (8.7%). Common suction devices include: wall suction (42.6%); floor suction connected to centralized suction (31.3%); portable table top suction (21.8%). Regarding, patterns of suction utilization in FANS: suction was reportedly used intermittently as needed to evacuate dust and fragments in 57.9%; 31.6% utilized suction throughout the procedure (Supplementary Table 2).

### Exit strategy and protocol for follow-up imaging (Table 2)

A total of 518 (76.2%) routinely place a double J stent postoperatively. The most common imaging modality (44.7%) remained X-ray KUB and/or ultrasound. A 24 h and 30-day NCCT were being advocated in 15.1% and 19.3% respectively.

**Table 2:** Exit strategy and management protocol following flexible ureteroscopy with FANS

	*N*	%
Exit strategy		
- Routine placement of double J stent	518	76.2%
- Placement of double J stent only if suspicion of injury, ureteral edema, residual fragments or intended re-intervention	100	14.7%
- Routine overnight ureteric catheter only	17	2.5%
- Overnight ureteric catheter only when no injury, residual fragments or extravasation of urine	25	3.7%
- Overnight ureteric catheter only in prestented patients only when no injury, residual fragments or extravasation of urine	20	2.9%
Hospital stay		
- Same day discharge (Ambulatory surgery)	128	18.8%
- Overnight observation < 24 h (hospital or insurance policy)	271	39.9%
- > 24 h (due to personal preference)	171	25.1%
- > 24 h (due to hospital or insurance policy)	110	16.2%
Usual postoperative follow-up imaging protocol		
- 24 h NCCT in all	103	15.1%
- NCCT within 72 h	54	7.9%
- 30 day NCCT in all	131	19.3%
- 24 h and 30 day NCCT in all	18	2.6%
- 3 month NCCT only	70	10.3%
- Xray and/or USG KUB at first visit and NCCT if needed	304	44.7%

### Challenges encountered in the utility of FANS and vision for future development (Table 3)

FANS is valued for improving stone-free rates (82.1%) and reducing sepsis (70.0%). Biggest challenges: suction-induced PCS collapse (51.9%) and lower pole access (37.6%).

**Table 3:** Challenges encountered in the utility of FANS and vision for future development

	*N*	%
Reasons for adopting FANS in one’s practice*		
- Achieve high single stage stone-free rate	558	82.1%
- Reduces sepsis and infection	476	70.0%
- Lowers intrarenal pressure and can do longer surgery safely	425	62.5%
- Good intraoperative visibility	389	57.2%
- Reduce reinterventions	387	56.9%
- Avoids unnecessary need for basketing	378	55.6%
- Tackle bigger >2 cm stones	371	54.6%
- Evidence is enough to use FANS	205	30.1%
- Peer pressure	112	16.5%
- Reduce postoperative need to place double J stents	103	15.1%
Most common challenge encountered during FANS flexible ureteroscopy*		
- Uncontrolled collapse of PCS due to suction	353	51.9%
- Ergonomically tiring to aspirate stones repeatedly	273	40.1%
- Lower pole access	256	37.6%
- Bleeding from PCS due to sheath movement	168	24.7%
- Difficulty removing multiple fragments	125	18.4%
- None	108	15.9%
- Scope malfunction due to manipulation	85	12.5%
- Sheath malfunction due to manipulation	78	11.5%
	36	5.3%
Most important future development area for FANS*		
- Miniaturization of sheaths and scopes	201	29.6%
- Automated irrigation, suction and pressure control	176	25.9%
- Defining irrigation suction parameters	132	19.4%
- Intrarenal pressure control mechanism	81	11.9%
- Ergonomics	68	10.0%
- Defining which FANS sheath to be used for the target stone volume	22	3.2%

*question allowed multiple answers

## Discussion

Our survey represents the largest global collaborative effort (EAU, PEARLS, IAU) on FANS utilization, with 680 participating surgeons. As FANS is a promising game-changer [7, 10], our findings help identify adoption hurdles and inform standardization in real-world practice.

### Rapid adoption and expanding indications

The survey underscores a remarkable shift in practice patterns. Compared to earlier reports where only 42.7% of urologists incorporated suction in fURS [4], our present survey revealed near-universal engagement: 91% of respondents now use some form of suction, and only 7.4% abstain from FANS entirely. Notably, 28.7% have replaced all conventional access sheaths with FANS in routine practice—a testament to its perceived utility. Survey respondents primarily value FANS for enhancing single-stage stone-free rates (82.1%) and reducing sepsis (70.0%). This aligns with growing evidence that suction mitigates key limitations of conventional fURS, including elevated intrarenal pressure(IRP), poor visibility, and residual fragments [8, 11–13].

FANS also expands the traditional boundaries of fURS. While most favoured FANS utilization in normal renal anatomy and adult population, its application now extends to larger stones. Respondents were split on the upper stone size threshold: 46.8% viewed 2 cm as the cutoff for single-stage FANS fURS, while 37.6% advocated for 3 cm. This change may be informed by emerging evidence, such as the recent multicenter randomized noninferiority trial by Zeng et al. [14], demonstrating non-inferiority stone-free rates (SFR) from FANS fURS compared to mini-PCNL for 2–3 cm stones. The preference for FANS in bilateral stones [15], lower pole [16], multiple calculi, and paediatric patients [17, 18] further highlights its role in complex scenarios previously dominated by percutaneous and/or staged approaches. FANS has also stretched operational time limits: while 45.4% adhered to a 90-minute time limit, 34.6% extended fURS surgeries to 120 min—indicating confidence in FANS’s safety profile but underscore more high level evidence and long term data to guide best practice and guidelines recommendation [19]. This also raises pertinent questions regarding thermal safety, ureteral injury risk, and sustained intrarenal pressure, which warrant dedicated study to define safe procedural limits.

### Technical heterogeneity and knowledge gaps

Despite enthusiasm, the survey exposes significant variability in techniques and knowledge gaps:

- **Optimal fluid dynamics**: Strikingly, 39.9% were unaware of their irrigation flow rates, and 28.1% did not know the exact suction pressure settings. This operational ambiguity reflects the pressing need for research, standardization and training regarding optimal fluid dynamics for lithotripsy and fragment clearance, informed by ratio of the endoscope-sheath diameter ratio (RESD), irrigation, suction and real-time IRP data [20].
- The most reported intraoperative hurdles by respondents were uncontrolled pelvicalyceal system (PCS) collapse due to suction (51.9%), tiring ergonomics for repeated stone aspiration(40.1%), and navigation to reach lower poles(37.6%). This “suction-induced collapse” can obscure visibility, induce decompression hematuria and complicate scope navigation. Such issues highlight the delicate balance required for optimal fluid dynamics—a challenge exacerbated by the lack of routine real-time IRP monitoring and standardized protocols.
- **Lack of standardization of procedure**: Despite established evidence of FANS, when used safely, maintains anatomical integrity as seen in the multicenter observation study with follow up to 3months [21], our survey still reflects diverse practice in the procedural steps(Figure.3 A). The introduction of a standardized protocol can help mitigate risk during generalization and widespread technology adoption.

### Equipment preferences and future directions

Disposable scopes(54.1%) of 7.5Fr(41.7%) paired with smaller 10/12Fr sheaths (42.4%) were adopted, reflecting a trend toward equipment miniaturization. There is also drastic decrease in the need for routine basket utilization, reserving basket need for stone relocation in the lower pole instead of fragment retrieval.

For future innovation, respondents prioritized:

1. **Defining irrigation/suction parameters** (19.4%), particularly automated systems optimizing pressure/flow in real-time settings.
2. **Integrated pressure control mechanisms** (25.9%) to prevent PCS collapse.
3. **Miniaturization** of instruments(29.6%) for pediatric and anatomically constrained cases.

The call for “suction-irrigation ratio (SIR) optimization” underscores the need for fluid dynamics research to perfectly orchestrate the “quadrifecta” of fURS: irrigation, suction, low/optimal IRP and thermal safety [22].

The barriers to the adoption of suction technology in endourology—including the cost of new devices, lack of availability, and lack of procedural standardization and training—must be continuously addressed, as highlighted in our previous work [4]. The global nature of this current survey underscores how these barriers are not uniformly distributed; disparities in equipment availability and training infrastructure across different geographic regions are a crucial contextual factor that likely influences both the adoption of FANS and procedural outcomes.

### Limitations and clinical implications

This survey has limitations inherent to questionnaire-based studies, including recall bias, non-response bias, and the inability to correlate preferences with objective outcomes. Regional disparities, dictated by FANS local availability, may limit the global generalizability. Nevertheless, our findings provide crucial insights:

- **Training & standardization**: The technical heterogeneity and knowledge gaps signal an urgent need for structured training programs and best-practice protocols to minimize learning curves. Societies like EAU/IAU should develop guidelines addressing sheath insertion, irrigation/suction settings, and case selection.
- **Research priorities**: Prospective studies should quantify SIR thresholds, validate stone-size/volume cutoffs, and evaluate automated pressure-regulated platforms. The role of FANS in anomalous kidneys, pediatrics, and unstented ureters warrants further prospective long term studies.
- **Technology development**: Industry investment in “smart sheaths” with embedded IRP sensors for pressure/flow monitoring and ergonomic designs is imperative.

## Conclusion

Our survey exposes two sides of the same coin, while FANS procedure indications and its widespread adoption are proof of its potential as a game changer in flexible ureteroscopy, the lack of a standardised technical approach must be addressed through evidence and education to facilitate broader implementation by surgeons. The strength of the survey is that it clearly outlines these aforementioned observations in a real-world global practice. It serves to validate existing evidence and allows for more critical research and evidence generation. This EAU, PEARLS and IAU collaborative effort ultimately aims to optimize patient outcomes.

## Supplementary Information

Below is the link to the electronic supplementary material.

Supplementary Material 1

Supplementary Material 2

## Data Availability

The data that support the findings of this study are available from the corresponding author upon reasonable request.
