# Supplementary material for: The global flexible and navigable suction ureteral access sheaths (FANS) survey on utility, practices and future needs in flexible ureteroscopy: a EAU Endourology, PEARLS and IAU collaboration
Source: World J Urol. 2026 Mar 10;44(1):229. doi: 10.1007/s00345-026-06321-4 (PMC12975862; doi:10.1007/s00345-026-06321-4)
Supplement: Supplementary file 1 — Supplementary Material 1 [file 345_2026_6321_MOESM1_ESM.pdf]

The landscape of Flexible Ureteroscopy evolves with the use of Flexible and Navigable Suction Ureteral Access Sheath (FANS) as more surgeons gain experience with it. On behalf of a collaborative effort between YAU Endourology and IAU, we invite you to spare 5 mins to answer this survey on how and why you use FANS in your institution.
We sincerely thank you in advance for sparing 5 minutes of your precious time.

Section 1. Demographics

We would like to learn more about you and your practice.

* 1. What is your gender?

- ◯ Male
- ◯ Female

* 2. How old are you?

- ◯ <30
- ◯ 31-40
- ◯ 41-50
- ◯ >50

* 3. Where do you practice?

* 4. The health system I work in is:

- ◯ Private/ corporate hospital
- ◯ University teaching hospital with residents
- ◯ Public hospital/ no residents
- ◯ I work part time in both Public and Private

* 5. I am:

- ◯ Consultant
- ◯ Fellow
- ◯ Resident

* 6. Do you have a endourology fellowship?

- ◯ Yes
- ◯ No

* 7. How many years of experience do you have as a urologist?

- <10
- 11-20
- >20
- In training

* 8. How Many RIRS procedures do you perform in your department yearly?

- <10
- 11-50
- 51-100
- 100-200
- >200

* 9. What percentage of RIRS utilise FANS?

- <25%
- 25-50%
- 50-75%
- Almost all
- NONE

* 10. What percentage of RIRS utilise conventional ureteral access sheath?

- <25%
- 25-50%
- 50-75%
- Almost all
- NONE

* 11. How many of your RIRS procedures are done using suction?

- <25%
- 25-50%
- 50-75%
- Almost all
- NONE

* 12. What percentage of RIRS that utilised conventional ureteral access sheath have been converted to FANS?

- <25%
- 25-50%
- >50-75%
- Almost all
- None

* 13. How many cases of Flexible and Navigable Suction Ureteral Access Sheaths (FANS) ureteroscopy does your department perform monthly?

- <10
- 11-15
- 16-20
- >20
- >50

* 14. How many cases of Flexible and Navigable Suction Ureteral Access Sheaths (FANS) ureteroscopy do YOU perform as a primary surgeon or assist (as a first assistant) monthly?

- <5
- 6-10
- 11-20
- >20
- >50

* 15. Do you routinely need an assistant during flexible ureteroscopy using FANS?

- none
- 1 assistant
- More than 1 assistant

16. Purpose of assistant (if required)

- ◯ Help stabilise sheath
- ◯ Help suction by regulating the suction vent
- ◯ Free text

* 17. What is your antibiotic regime used? (Tick all that applies)

- [ ] Intraoperative single dose antibiotics in all and treat any positive culture
- [ ] Treat only if culture positive
- [ ] Postoperative antibiotics only if required
- [ ] Mandatory routine postoperative antibiotics

Section 2. Indication for FANS

We would like to now how and where FANS fit in your armamentarium of endourological urolithiasis treatment.

* 18. Your Most common indications for FANS? choose all that apply

- [ ] renal stone in any location
- [ ] renal stone in upper pole only
- [ ] renal stones in mid pole only
- [ ] renal stones in lower pole only
- [ ] renal pelvic stone only
- [ ] multiple renal stones in multiple locations or single location only
- [ ] stone diameter > 1 cm
- [ ] stone diameter >1cm <2cm
- [ ] any stone diameter
- [ ] stone volume <1000 mm3
- [ ] stone volume>1000mm3 <2000 mm3
- [ ] any stone volume
- [ ] proximal ureteric stone only
- [ ] partial staghorn only
- [ ] Bilateral renal stones

* 19. In which age group do you use FANS? (check all that applies)

- [ ] adult 18 years < 70 years only
- [ ] adult >18 years of any age if feasible
- [ ] children above 5 years up to 17 years only
- [ ] children 1-5 years including Infants

* 20. You will perform FANS only in: (all that apply to your practice)

- [ ] Normal renal anatomy only
- [ ] Anomalous kidney if stone accessible only
- [ ] Pre-stented only
- [ ] Any ureter that allows easy placement of ureteral access sheath

* 21. What is your upper limit of stone size in single stage RIRS?

- [ ] 2cm
- [ ] 3cm
- [ ] 4cm or above
- [ ] 2000mm3
- [ ] 3000mm3

Section 3: Peri-operative preparation and strategies

We would like to understand your routine practices in FANS, including procedural steps, FANS sheath size, flexible ureteroscope sizes and types, irrigation and suction methods and settings, laser choices and settings, need for basket accessories.

* 22. My standard steps in FANS flexible ureteroscopy include:
(Please choose all the steps that you do in your routine practice)

- [ ] Ureteric catheter placement and Retrograde Pyelogram
- [ ] Semirigid ureteroscopy to assess ureter
- [ ] Double lumen catheter to assess elasticity
- [ ] Placement of 2 wires in ureter. 1 for safety and 1 for UAS insertion
- [ ] Placement of 1 wire only and UAS Insertion and removal of safety wire
- [ ] Insertion of scope

23. My preferred FANS sheath is:

- ○ 10/12 Fr
- ○ 11/13 Fr
- ○ 12/14 Fr
- ○ No fixed choice
- ○ Bigger stones bigger sheath
- ○ Others

[ ]

* 24. My usual Scope for FANS is: (choose all that apply)

- [ ] Reusable only
- [ ] Disposable only
- [ ] Depends on stone location and stone volume
- [ ] Depends on scope availability
- [ ] 7.5Fr mostly
- [ ] >=8Fr mostly
- [ ] 7.5 Fr for smaller sheath size (10/12 Fr and 11/13 Fr FANS) and 8 Fr and above for bigger sheath size (12/14Fr)
- [ ] Intrarenal pressure monitoring flexible ureteroscopes

* 25. My surgery is performed under:

- ◯ General anaesthesia, no respiratory control
- ◯ General anaesthesia, with respiratory control (apnoea Or gated respiration)
- ◯ Spinal anaesthesia only
- ◯ Local anaesthesia with light sedation

* 26. My preferred irrigation technique when using FANS is:

- ◯ Gravity Only
- ◯ Gravity with pressurised bag only
- ◯ Endomat pump
- ◯ Endoflow (Traxer flow From Rocamed)
- ◯ Automated intrarenal pressure and irrigation flow pumps
- ◯ Other vacuum based pumps
- ◯ If other: Free text specify

____

* 27. My usual irrigation flow rate settings (in ml/min)

- [ ] 40ml/min
- [ ] 50ml/min
- [ ] 100ml/min
- [ ] input value: free text __ml/min
- [ ] 150ml/min
- [ ] 200ml/min
- [ ] I can't measure flow rate so anything goes.

____

* 28. My suction apparatus is:

- ◯ Wall suction
- ◯ Floor suction
- ◯ Portable table top suction
- ◯ Other (please specify)

____

* 29. I Use Suction:

- ◯ Throughout the procedure
- ◯ Intermittently as needed to evacuate dust and fragments
- ◯ Only at the end to evacuate dust and fragments
- ◯ I only depend on passive fluid evacuation and no suction.

* 30. My usual suction level is

- ◯ 50mmHg
- ◯ 100mmHg
- ◯ 150mmHg
- ◯ 200mmHg
- ◯ I just put it on maximum
- ◯ I have no idea
- ◯ Other (please specify value and unit) eg 180mmHg/ 0.02mPa

* 31. My laser choices for FANS are:

- ☐ TFL (any brand)
- ☐ Low power Holmium (<40W)
- ☐ High power Holmium (>40W)
- ☐ Thulium-YAG laser
- ☐ MOSES or Vapor tunnel mode laser
- ☐ MAGNETO HOLMIUM laser

* 32. My usual lasing strategy is:

- ◯ Dust mostly, then fragment and aspirate
- ◯ Dust only and aspirate
- ◯ Fragmentation and evacuation only
- ◯ Based on how the stone behaves at initial lasing

* 33. I use a Basket:

- ◯ Only to extract fragments
- ◯ Never, as I can aspirate fragments
- ◯ Always to relocate lower pole stones for fear of scope / sheath damage
- ◯ Only if I cannot reach lower pole stones at all

* 34. When utilising FANS, (provided that the surgery was uneventful and patient was stable throughout the procedure), my upper time limit of operation time is:

- ◯ 90 mins
- ◯ 120 mins
- ◯ 150 mins
- ◯ 180 mins
- ◯ >180 mins

Section 4: Exit strategy and Imaging

We would like to understand your choice of exit strategy and type and time point of imaging following FANS flexible ureteroscopy.

* 35. After complete lithotripsy I always:

- ◯ Always to visualise the pelvicalyceal system (PCS) and remove dust and fragments and inspect for injuries
- ◯ Visualise the PCS to remove any fragments only, Dust I'm OK to leave behind
- ◯ Visualise the PCS only if I suspect injury or residual fragments remaining
- ◯ Do not routinely visualise but just do a contrast study to document no injury and filling defects
- ◯ Don't do any visual inspection or contrast as a routine
- ◯ Don't use fluoroscopy at all

* 36. After complete lithotripsy I always:

- ◯ Place a double J stent as routine only
- ◯ Place a double J stent if suspicion of injury, ureteral edema, residual fragment (RF) present or intended re-intervention.
- ◯ Place an overnight ureteric catheter only in all cases.
- ◯ Place a Ureteric catheter in any patient as overnight Strategy Only when NO INJURY/ NO RF remains / NO extravasation of urine
- ◯ Ureteric catheter in prestented patients only when there is NO INJURY/ NO RF remains / NO extravasation of urine

* 37. My patients undergoing FANS flexible ureteroscopy:-

- ◯ Same day discharge (Ambulatory surgery)
- ◯ Overnight observation <24 hour (hospital or insurance policy)
- ◯ >24 hour (due to my personal preference)
- ◯ >24 hour (due to hospital or insurance policy)

* 38. My usual imaging evaluation post op is NCCT is:

- ◯ 24 hr NCCT in all
- ◯ After 24hr NCCT within 72 hour
- ◯ 30 day NCCT in all
- ◯ 24 hr and 30 day NCCT in all
- ◯ 3 month NCCT only
- ◯ XRAY and/or USG KUB at first visit and if needed NCCT.

Section 5: Your view on FANS

We would like to know your view on the utility and future of FANS.

* 39. In my practice, FANS is my preferred way for RIRS
Because: Choose all that apply

- ☐ achieve high single stage SFR
- ☐ reduces sepsis and infective complications
- ☐ reduce re-interventions
- ☐ do bigger > 2cm Stones
- ☐ peer pressure
- ☐ evidence is enough to use FANS
- ☐ vision is very good during RIRS
- ☐ avoids unnecessary need for BASKETING
- ☐ intrarenal pressure is low and I can do longer surgery safely
- ☐ reduced my postoperative need to place double J stents

* 40. Most common challenge I encounter during FANS flexible ureteroscopy: (choose all that apply)

- [ ] Constant bleeding from The PCS, due to sheath movement
- [ ] Too fast collapse of the PCS due to suction
- [ ] Lower pole access
- [ ] Difficulty removing multiple fragments
- [ ] Ergonomically Tiring to repeat stone aspiration multiple times
- [ ] Sheath malfunction due to manipulation
- [ ] Scope malfunction due to manipulation
- [ ] Other (please specify)
- [ ] None of the above

* 41. In my practice, I think the single most important FUTURE development for FANS is :

- ○ Defining irrigation suction parameters
- ○ Smaller Sheaths and Smaller Scopes
- ○ IRP control mechanism
- ○ Automated irrigation, pressure and suction control
- ○ Ergonomics
- ○ Defining which FANS sheath in which stone volume

On behalf of EAU-YAU Endourology and IAU - we thank you taking your time in completing this survey! Please press DONE at the very end to save your survey response!
