# Supplementary material for: The global flexible and navigable suction ureteral access sheaths (FANS) survey on utility, practices and future needs in flexible ureteroscopy: a EAU Endourology, PEARLS and IAU collaboration
Source: World J Urol. 2026 Mar 10;44(1):229. doi: 10.1007/s00345-026-06321-4 (PMC12975862; doi:10.1007/s00345-026-06321-4)
Supplement: Supplementary file 2 — Supplementary Material 2 [file 345_2026_6321_MOESM2_ESM.docx]

Supplementary Table 1. Geographical distribution of survey participants (by country)

| Country   - China - Turkey - India - Egypt - Phillipines - Indonesia - Singapore - Brazil - United Kingdom - Malaysia - Italy - Russia - Spain - Greece - Trinidad and Tobago - Morocco - Poland - Qatar - United States - Uzbekistan - Japan - Nepal - Saudi Arabia - Argentina - France - Mexico - Taiwan - Romania - Switzerland - Armenia - Belgium - Bulgaria - Central Africa - Kuwait - Liberia - North Macedonia - Pakistan - Serbia - Sweden - Thailand - United Arab Emirates - Austria - Canada - Chile - Colombia - Georgia - Iraq - Ireland - Norway - Peru - Portugal - South Korea | N 263 74 54 31  23 22  20  17  17  14  13  8  8  7  7  6  6  6  6  6  5  5  5  4  4  4  4  3  3  2  2  2  2  2  2  2  2  2  2  2  2  1  1  1  1  1  1  1  1  1  1  1 |
| --- | --- |

Supplementary Table 2. Irrigation and suction devices utilization

| Preferred irrigation device when using FANS   - Gravity only - Gravity with pressurized bag only - Endomat pump - Endoflow (Traxer flow from Rocamed) - Automated intrarenal pressure and irrigation flow pumps - Other vacuum based pumps - others | 128  165 107 77 59  96 48 | 18.8% 24.3% 15.7% 11.3% 8.7%  14.1% 7.1% |
| --- | --- | --- |
| Suction apparatus   - Wall suction - Floor suction - Portable table top suction - others | 290 213 148 29 | 42.6% 31.3% 21.8% 4.3% |
| Suction is utilized   - throughout the procedure - intermittently as needed to evacuate dust and fragments - only at the end to evacuate dust and fragments - only depend on passive fluid evacuation and no suction | 215 394  48  23 | 31.6% 57.9%  7.1%  3.4% |
